# Hypoxia and TGF-β1 lead to endostatin resistance by cooperatively increasing cancer stem cells in A549 transplantation tumors

**DOI:** 10.1186/s13578-015-0064-4

**Published:** 2015-12-23

**Authors:** Yuyi Wang, Ming Jiang, Zhixi Li, Jiantao Wang, Chi Du, Liu Yanyang, Yang Yu, Xia Wang, Nan Zhang, Maoyuan Zhao, Li Wang, Mei Li, Feng Luo

**Affiliations:** Department of Medical Oncology, Cancer Center and State Key Laboratory of Biotherapy, West China Hospital of Sichuan University, Chengdu, Sichuan 61004 People’s Republic of China; Department of Oncology, The Second People’s Hospital of Neijiang, Neijiang, Sichuan 641000 People’s Republic of China

**Keywords:** Endostatin, Lung cancer stem cells, Hypoxia, TGF-β1

## Abstract

**Background:**

Lung cancer is the leading cause of cancer-related deaths worldwide, and treatments for lung cancer have a high failure rate. Anti-angiogenic therapy is also often ineffective because of refractory disease. Endostatin (ES) is one of the most widely-used anti-angiogenic drugs for lung cancer in China, and resistance to it is a barrier that needs to be resolved. It has been shown that myeloid-derived suppressor cells (MDSCs) are involved in resistance to ES. Whether other cells and/or cell factors in the tumor microenvironment that have been shown to be related to resistance to other anti-cancer drugs are also involved in ES resistance is unknown.

**Results:**

In this study, we showed that after continuously treatment with ES for 12 days, volumes of A549 transplantation tumors of mice reached the sizes of tumors which were borne by mice that were treated with normal saline and this meant that resistance to ES appeared. Cancer stem cells (CSCs), which have been widely accepted as one of reasons responsible for resistance to many anti-tumor drugs were also being discovered increased proportionally in A549 transplantation tumors after ES treatment for 12 days. During further exploration of reasons for this increase, we discovered that after ES treatment, microvessel density and vascular endothelial growth factor level was decreased in tumors, whereas transforming growth factor (TGF)-β1 level was elevated, and MDSCs, one of the sources of TGF-β1, were also increased. We speculate that hypoxia and TGF-β1 are responsible for the increased CSC number in A549 transplantation tumors. By using cobalt chloride to mimic hypoxia and human recombinant TGF-β1 in vitro, we found that hypoxia and TGF-β1can indeed enhance the stemness of A549 cells. In addition, the inductive effect of hypoxia is stronger than TGF-β1, and the combination of both is stronger than either alone, which is first report of such a finding, to our knowledge.

**Conclusions:**

Increased TGF-β1 and strengthened hypoxia in A549 transplantation tumors, as a result of ES therapy, cooperatively increase proportion of CSCs that are involved in ES resistance which was revealed by failure of tumor volume repression after continuously treatment with ES for 12 days.

## Background

Currently, lung cancer is the leading cause of morbidity and mortality from cancer, accounting for 30 % of all cancer-related deaths [1]. Although major steps forward in the development of a cure for lung cancer have been made, many patients still die within a few years after diagnosis. Among these cures, anti-angiogenic therapies have been found to be relatively short-lived, and most patients treated with it eventually relapse and progress. The primary explanation for this failure is considered to be an inherent or acquired resistance to this type of therapy [2].

Endostatin (ES) is a widely-used endogenous, broad-spectrum, anti-angiogenic inhibitor, and has been shown to be clinically effective in China. A number of independent studies researching into the mechanisms underlying the anti-tumor activities of ES have shown that the drug restrains tumor growth through various channels, including repressing combination between vascular endothelial growth factor (VEGF) and endothelial cells (ECs), initiating EC apoptosis [3], and bonding to cell surface receptors such as nucleolin [4] to regulate a myriad of signaling cascades. Recently, ES was found to be an inhibitor of androgen receptor and could be a new choice for castration-resistant prostate cancer [5], and it is also a suppressor of basic fibroblast growth factor-induced angiogenesis in melanoma [6]. However, we have recently reported resistance to ES in tumor-bearing mice [7], therefore, identifying the reason for this resistance in order to overcome-this problem is important. We have shown that CD11b^+^Gr1^+^ myeloid-derived suppressor cells (MDSCs) are involved in resistance to ES [8], but further studies are needed to identify whether there are other underlying reasons as well.

Recent research has shown that, both cells and cell factors, inside the tumor microenvironment are directly or indirectly involved in drug resistance, including cells such as T-regulatory cells (Tregs) [9], cancer stem cells (CSCs) [10], tumor-associated fibroblasts (TAFs) [11], and tumor-associated microphages (TAMs) [12], and cell factors such as transforming growth factor (TGF)-β1 [13], hypoxia-inducible factor (HIF)-1α [14], interleukin (IL)-17 [15], IL-6 [16], IL-8 [17], IL-10 [18], and tumour necrosis factor (TNF)-α [19]. Whether these cells or cell factors are also responsible for resistance to ES needs to be further studied. Primarily, CSCs, which have drawn extensive attention in recent years, should be particularly considered.

The CSC model was proposed in 1997, with the discovery of stem cells in leukemia [20], and evidence of the existence of lung CSCs (LCSCs) was shown by Giangreco in 2009 [21]. CSCs, which are also known as tumor-initiating cells and stem-like cancer cells, are a subpopulation of tumor cells that have been shown into have the properties of immortal production, self-renewal, and tumor formation in xenograft mouse models. CSCs have been shown to be involved in tumor growth, metastasis, and tumor relapse [22]. CSCs also taken part in drug resistance through a number of different mechanisms. For example, CSCs spend the majority of their time in the G0 phase of the cell cycle, thus anti-neoplastic drugs, which act on cycling cell populations, are inefficient against CSCs [23]. Conducting research on CSCs is important, and the first step in this is identification and separation of CSCs from other ‘ordinary’ tumor cells, which has been thoroughly researched. For some kinds of leukemia, widely acceptable protein markers have been discovered, but it is still unclear which markers are the best for lung cancer. The majority of studies have shown that CD133^+^ cells, ALDH^+^ cells, and SP (side population) cells contain many more stem cell-like cells, therefore, these three cell markers are usually used to identify and sort LCSCs.

We speculated that CSCs are involved in resistance to ES, and that treatment with ES will lead to an increase of CSCs in lung cancer, resulting in resistance. This study aimed to assess the numbers of CSCs in lung cancer after treatment with ES and explore reasons of this change.

## Results

### ES increased the proportion of cancer stem cells in A549 transplantation tumors

**Fig. 1:**
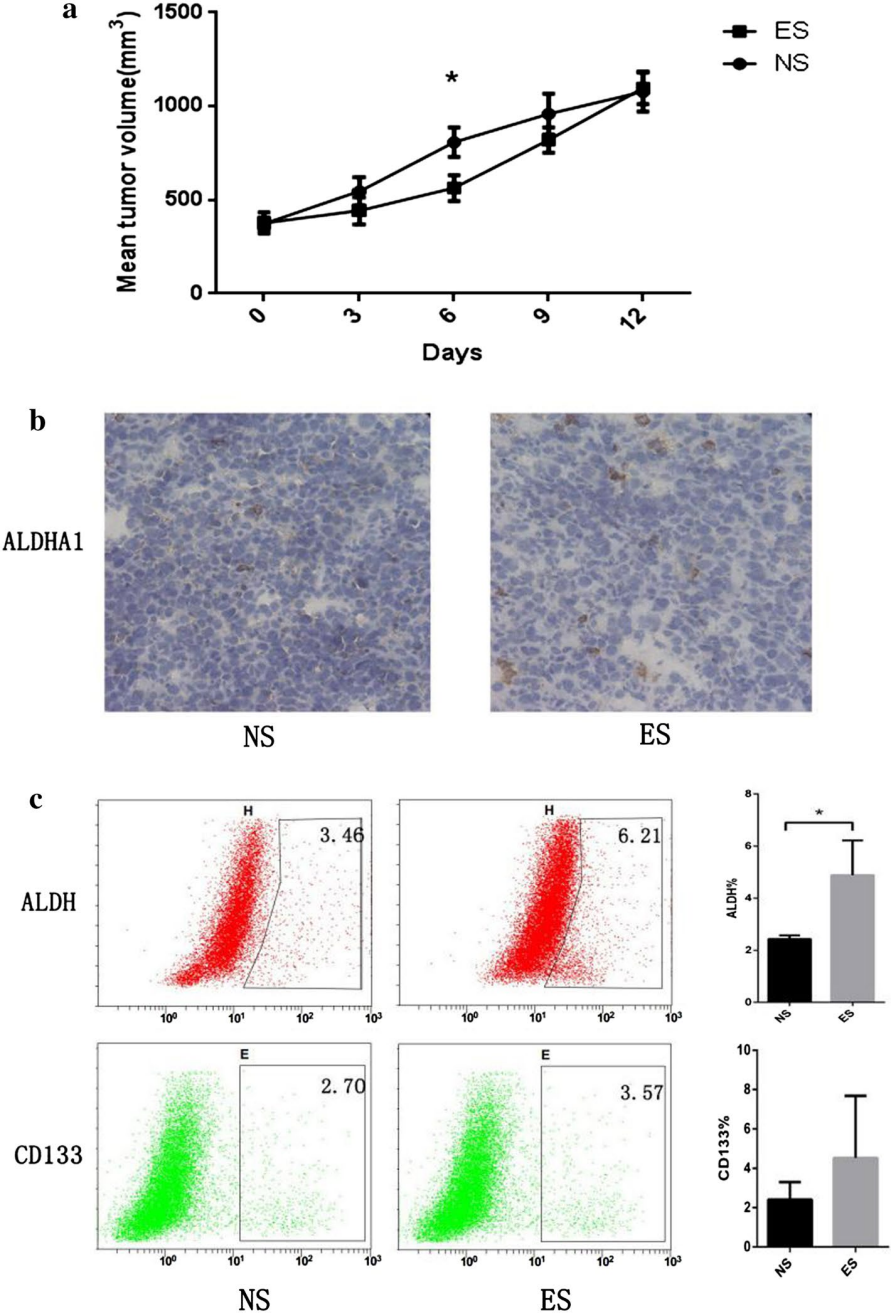
The proportion of cancer stem cells in A549 transplantation tumors after 12 days of treatment. **a** Change in tumor volumes after endostatin (ES) or normal saline (NS) treatment. **b** ALDHA1 expression in ES and NS groups. **c** Flow cytometry showing that ALDH-positive rate was higher in the ES group. *P < 0.05 compared with NS group. CD133 positive cell amount is also higher in ES group and the difference does not reach statistical significance

FACS and IHC were used to assess the difference in the expression of CSC-related protein (ALDH and CD133) between the NS and ES groups. Considering that ALDH or CD133 is feasible being used as protein markers to identify LCSCs in A549 cells has been shown by large amounts of studies [24–30], We also chosen ALDH and CD133 as markers of CSC in our present study. Flow cytometry shown that there were more CSCs in the tumors in the ES group. IHC showed that the proportion of ALDH^+^ (*P* = 0.0292*) and CD133^+^ (*P* = 0.3265) cells were increased in the ES group compared with the NS group. There were also more ALDHA1^+^ (an isoform aldehyde dehydrogenase of ALDH) cells in tissue sections from the ES group (Fig. 1). Higher proportions of ALDH^+^, CD133^+^ and ALDHA1^+^ cells in ES group indicated that LCSCs increased after ES treatment.

### ES intensified hypoxia and improved secretion of TGF-β1 in A549 transplantation tumor

**Fig. 2:**
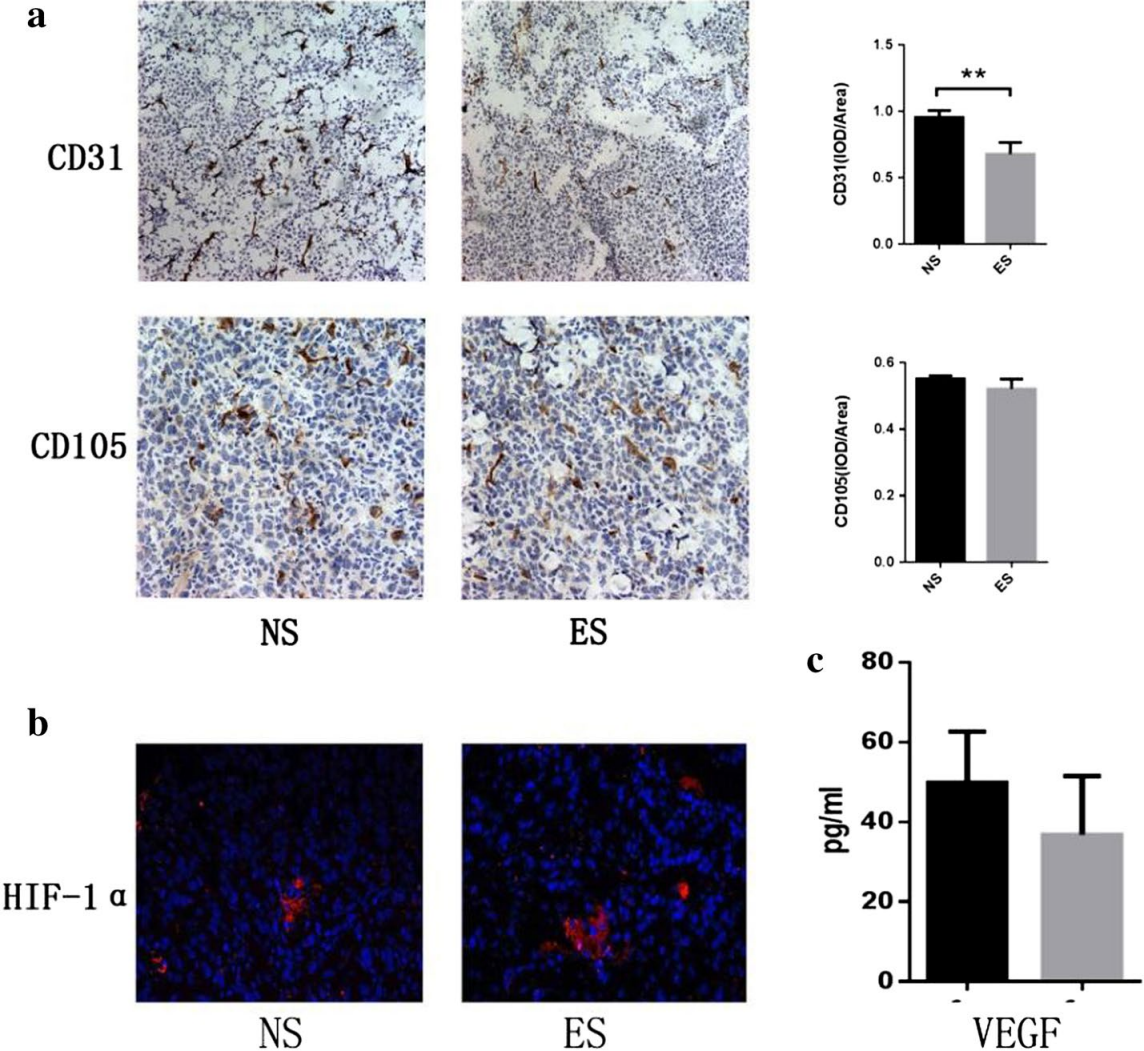
MVD, oxygen concentration, and VEGF level in A549 transplantation tumors after 12 days of treatment. **a** CD31 and CD105 expression in A549 transplantation tumors with endostatin (ES) or normal saline (NS) treatment for 8 days. Positive rate was lower in the ES than in the NS group. **P < 0.001 compared with NS group. CD105 expression was also lower in the ES group, although this was not statistically significant. **b** HIF-1α expression in ES and NS groups. **c** VEGF level was lower in the ES group, although this was not statistically significant

Anti-angiogenic agents work mainly by suppressing vascularization to decrease blood supply to the tumor and arrest its growth. In this present study, immunohistochemical expression of CD31 and CD105 were utilized to test changes of microvessel density (MVD) for CD31, as we all know, was expressed constitutively on the surface of ECs and is widely used to quantify MVD. CD105 was also chosen because it shown great specificity for the tumor vasculature, which is only expressed in the ECs of the tumor blood vessel, without being present in the normal ECs [31], and also was a prominent feature of newly formed blood vessels, but rarely expressed in pre-existing tumor vessels [32]. The IHC study showed that CD31^+^ and CD105^+^ cells were reduced in ES group, indicating that MVD declined in A549 transplantation tumors after ES treatment. VEGF (detected by VEGF ELISA kit) also decreased, whereas HIF-1α in the same tissue was shown by immunofluorescence to be increased (Fig. 2). These results agree with our previous studies [33]. Hypoxia is indeed involved in the induction of CSC, but whether it can directly enhance the stemness of A549 cells needs further study.

**Fig. 3:**
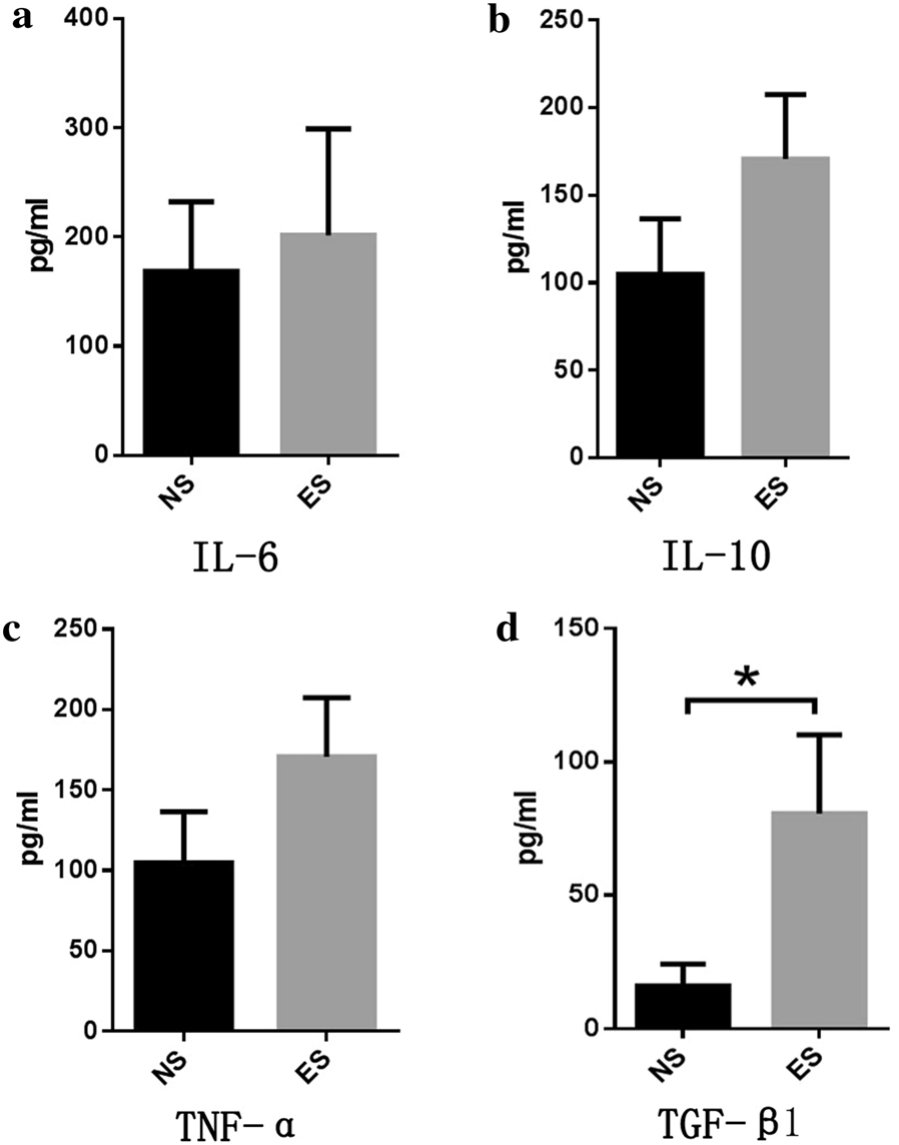
Levels of major inflammatory factors elevated after endostatin treatment: **a** IL-6, **b** IL-10, **c** TNF-α and **d** TGF-β1. *P < 0.05 compared with NS group

To explore more elements relevant to increased LCSC, we detected some major cell types and inflammation factors in A549 transplantation tumor tissues, and discovered that TGF-β1 was significantly increased (*P* = 0.0217*). Other factors, including TNF-α, IL-6, and IL-10, were also increased, although these increases were not statistically significant (Fig. 3). We speculate that TGF-β1 may be connected with the increase in CSCs in the ES group, but further studies are required to confirm this.

### TGF-β1 and hypoxia cooperatively increased CSCs

We further assessed whether hypoxia and TGF-β1 take part in CSC induction directly, and whether they act independently or cooperatively. CoCl_2_, which is frequent-used and widely accepted as chemical hypoxia-induced agent to mimic hypoxia [34–36] was applied to mimic hypoxia in vitro. We treated A549 cells with CoCl_2_, with the human recombinant TGF-β1, and with the combination of both TGF-β1 and CoCl_2_. We found that both hypoxia and TGF-β1 could enhance the stemness of A549 cells. The induction effect of hypoxia was stronger than that of TGF-β1, and when both were combined, the effect was stronger still, implying that there is crosstalk between hypoxia and TGF-β1.

**Fig. 4:**
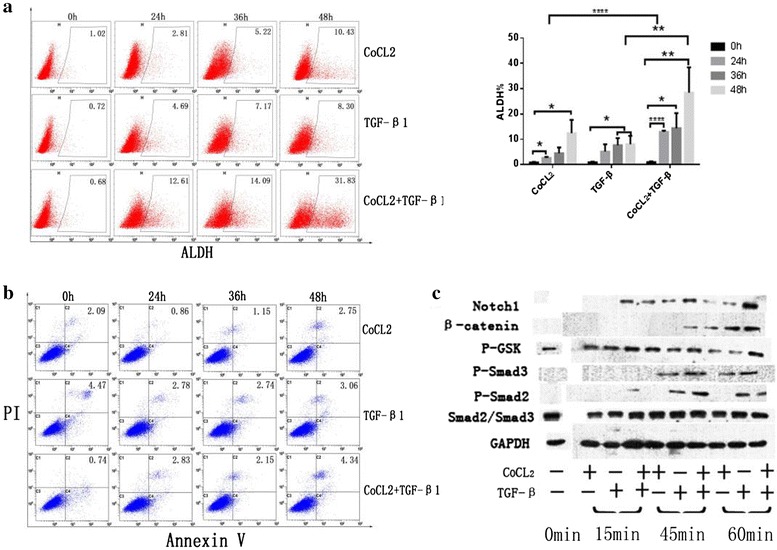
Effects of hypoxia and TGF-β1 on the ALDH-positive cell proportion of A549 cell cultures. **a** Hypoxia, TGF-β1, or the combination of both all led to a higher ALDH-positive proportion. Induction by hypoxia was stronger than that by TGF-β1, and the combination was stronger than either factor alone. *P < 0.05, **P < 0.01, ****P < 0.001. **b** Hypoxia and TGF-β1 did not cause apoptosis of A549 cells at 24, 36, or 48 h. **c** Protein expression of the related signaling pathway proteins Notch1, P-Smad2, and P-Smad3 were elevated

**Fig. 5:**
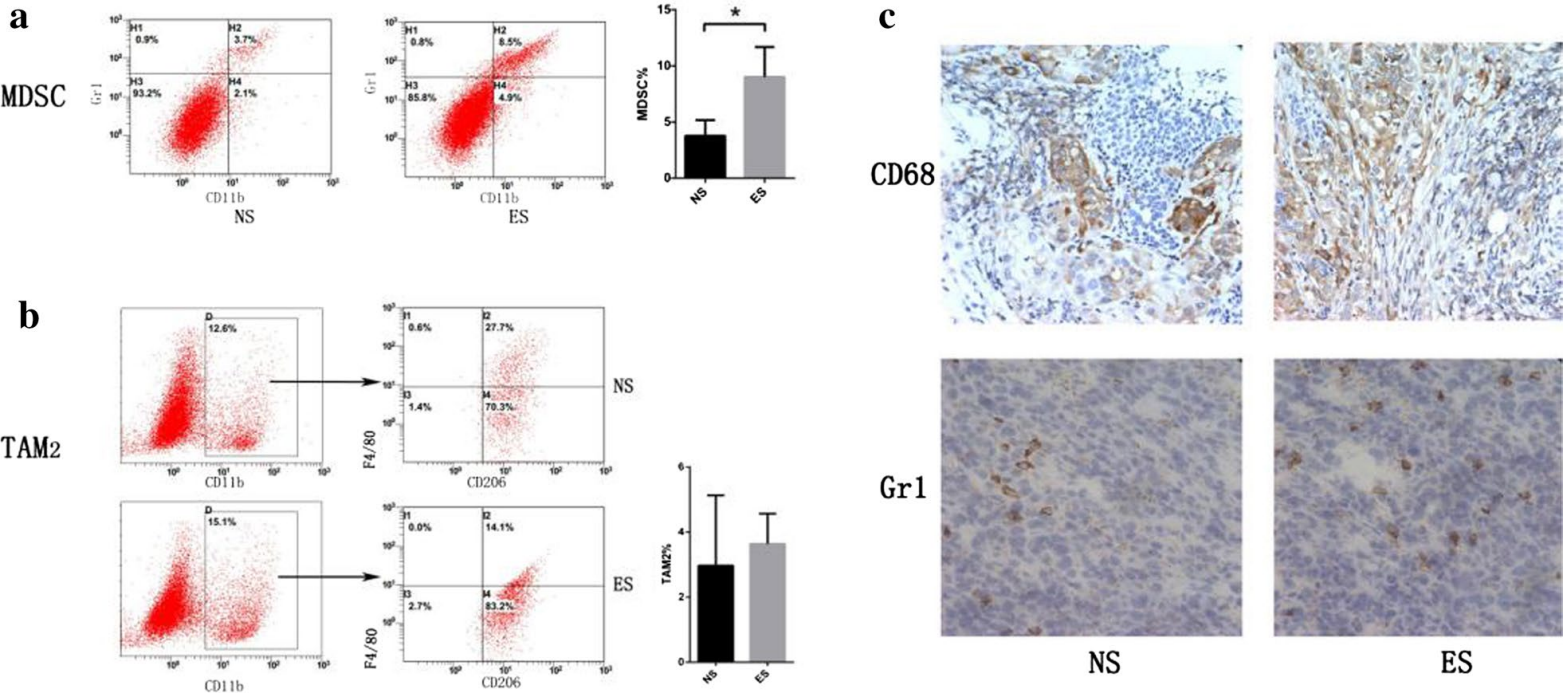
Endostatin treatment increased tumor-associated macrophages in A549 transplantation tumors. **a** Flow cytometry showed an increase in MDSCs in the ES group; *P < 0.05 compared with the NS group. **b** TAM2 in the ES group also increased, although this difference did not reach statistical significance. **c** Immunohistochemistry results showed that CD68 and Gr1 expression was stronger in the ES group compared with the NS group

We further measured the amount of protein exchange in several commonly accepted signaling pathways concerning CSCs and in Smad pathways after treatment as above, and the result showed that expression of Notch1, β-catenin, P-Smad2, and P-Smad3 were all elevated (Figs. 4, 5).

### MDSCs, and TAM2 were increased in A549 transplantation tumor after ES treatment

We assessed whether MDSC and TAM also rose after ES treatment of A549 transplantation tumor in NOD/SCID mice. We assessed TAM2 rather than TAM because of its closer relationship with immune suppression in tumors. In addition, as two of the major sources of TGF-β1, MDSC and TAM2 may induce CSCs indirectly. Our study showed that both CD11b^+^Gr1^+^MDSCs (*P* = 0.0026**) and CD11b^+^F4/80^+^CD206^+^TAM2 (*P* = 0.6475) increased in the ES group compared with the NS group.

## Discussion

In this study, we showed that after continuously treatment with ES for 12 days, volumes of A549 transplantation tumors of mice reached the sizes of tumors which were borne by mice that were treated with normal saline (NS) and this meant that resistance to ES appeared. CSCs, which have been widely accepted as one of reasons responsible for resistance to many anti-tumor drugs were also being discovered increased proportionally in A549 transplantation tumors after ES treatment for 12 days. In addition, intensified hypoxia, and promoted TGF-β1secretion were also observed. To further explore the relationship between CSCs, hypoxia, and TGF-β1, we assessed the effect of hypoxia and TGF-β1 on A549 cells in vitro, and verified that hypoxia and TGF-β1 are capable of inducing CSCs independently, as the proportion of CSCs in A549 cells increased when they were treated with CoCl_2_ and recombinant human TGF-β1 separately. We also found that treatment with both hypoxia and TGF-β1 enhanced CSCs induction, which was related to greater activation of Notch1, Smad2, and Smad3 in A549 cells. Finally, we found that MDSCs, a source of TGF-β1, increased in the A549 transplantation tumor model, and this might be one explanation for elevation of TGF-β1 level in tumors.

Research in recent years has shown that tumor stem cells are responsible for failure of anti-angiogenesis treatment in cancer. Metastasis is one of the reasons for drug resistance. Studies have shown that the number of CSCs closely correlates with tumor metastasis [37], with more CSCs being found in lymph node metastases than in primary tumors [38]. CSCs are also involved in angiogenesis and lymphangiogenesis because they express angiogenic and lymphangiogenic factors. Furthermore, CSCs can even differentiate into ECs to promote metastasis of cancers. One study found that when culture on Matrigeld in the presence of VEGF, breast tumor stem cells could turn into ECs and organize into capillary-like structures after 6 h of culture [39]. All of these phenomena are related to the complex tumor microenvironment, as many of its components, such as IL-6, HIF-1α, and TGF-β, are concerned with induction and maintenance of CSCs. Hypoxia is especially important, and is an independent prognostic factor correlated with advanced stages of malignant tumors. As an essential feature of the tumor microenvironment, hypoxia is vital in drug resistance. Studies have shown that one of the mechanisms of resistance is the capability of hypoxia to inhibit tumor cell differentiation and promote maintenance of CSCs [40].

TGF-β is a member of the TGF-β superfamily. It is well known that TGF-β plays a dual role in tumorigenesis, acting as a suppressor during tumor initiation or in the early stages, but as a promoter during cancer progression and metastasis in the more advanced stages. TGF-β has also been shown to be involved in immune suppression, epithelial–mesenchymal transition (EMT) promotion, and angiogenesis induction. In addition, TGF-β has been shown to stimulate the differentiation of mesenchymal precursors into myofibroblasts and thus contribute to the generation of CAFs. Furthermore, TGF-β also plays an important role in induction and maintenance of CSCs. Mani et al. found that TGF-β signaling was necessary for maintenance of the stem cell-like properties and tumorigenic activity of tumor cells through induction of EMT. TGF-β1 and versican are involved in the molecular mechanism of tumor sphere formation TGF-β1 is a triggering molecule and stimulates versican [41], and our study also showed that versican is involved in resistance to ES [7]. A close relationship exists between hypoxia and CSCs and many studies have shown that intratumoral hypoxia enhances cell stemness in various cancers, including lung adenocarcinomas [42], laryngeal cancers [43], pancreatic cancers [44], and glioblastomas [45]. There are a number of possible underlying mechanisms. First, hypoxia promotes activation of genes and increases levels of proteins involved with stem cells, such as activation of insulin-like growth factor 1 receptor [46], HIF-1α [47], and the *DLK1* gene [48], and up-regulation of OCT3/4 and SOX2 [49]. Secondly, hypoxia can affect CSCs through its impact on CSC niches. Hypoxia plays an important role in the composition of tumor-associated stromal cells and the evolution of tumor stroma, and leads to enrichment of undifferentiated stromal cells, which may provide a favorable microenvironment for maintaining tumor cells in a stem cell state. Finally, hypoxia also regulates expression of paracrine factors by ECs and other cells for the maintenance of CSCs [50]. Hypoxia should be considered when studies on tumor improvement are being conducted.

Utilization of anti-angiogenesis drugs will lead to intensified hypoxia, and increase in cell factors and inflammation factors, such as TGF-β1. TGF-β1and hypoxia have been shown to be involved separately in resistance to different kinds of anti-tumor agents. However, there are only a few studies reporting on whether these two factors act cooperatively, and for more detailed aspects, such as anti-angiogenesis drug-resistance, the effect of the combination has not been assessed. According to some studies, a close link exists between hypoxia and TGF-β1, and the effect they have on tumor promotion mainly includes three aspects. (1) Immunosuppression: hypoxia can promote the release of TGF-β1 by inducing IL-19 secretion [51, 52], and TGF-β1 derived from induction of hypoxia will increase the proportion of T-regs, resulting immunsuppression [53]. (2) Metastasis: in breast cancer, hypoxia and TGF-β1work synergistically to promote secretion of VEGF and stromal derived factor 1 receptor (CXCR4), and inhibition of either the HIF-1α or the TGF-β pathway in tumor cells was shown to decrease bone metastasis and improve survival rates, with no further effect of double blockade, while, unlike molecular blockade, combined drug treatment decreased bone metastases more than either alone [54]. Hypoxia can also improve secretion of TGF-β1 from mesenchymal stem cells to increase the invasiveness of breast cancer cells [55]. (3) CSCs: hypoxia and TGF-β1 improve transcription and maintain stemness of hematopoietic stem cells by acting on the *CDKN1C/p57* gene, and hypoxia can increase the sensitivity of hematopoietic stem cells to TGF-β1. The cell cycle pause, an important property of stem cells that is induced by TGF-β1, relies on hypoxia [56]. In the current study, we found that hypoxia and TGF-β can also induce CSCs formation cooperatively, which is the first such report, to our knowledge.

Along with MDSCs, TAM is a source of TGF-β1. TAM induces tumor cells turning into CSCs by secretion of TGF-β1 [57]. MDSCs can produce various immunosuppressive factors, including TGF-β1 [58]. In tumor-bearing mice, MDSC can express membrane-bound TGF-1 to suppress anti-tumor immunity [59].

In this study, we have shown that ES can increase CSC formation, and increased numbers of CSCs are one of the reasons for ES resistance which was revealed by failure of tumor volume repression after continuously treatment with ES for 12 days. To be more detailed, ES strengthens hypoxia in tumor tissue and improves secretion of TGF-β1by activating Smad2, Smad3, β-catenin and Notch1. Hypoxia and TGF-β1 increase CSC induction. We also found that MDSC, a source of TGF-β1, was increased in A549 transplantation tumors.

## Conclusions

Our results indicate that increased TGF-β1 and strengthened hypoxia in A549 transplantation tumors, as a result of ES therapy, cooperatively increase proportion of CSCs which are involved in ES resistance which was revealed by failure of tumor volume repression after continuously treatment with ES for 12 days.

## Methods

### Tumor cell culture and treatment

The A549 cell line was obtained from the American Type Culture Collection (ATCC, Manassas, VA, USA). Cells were cultured in Dulbecco’s modified Eagle’s medium (Life Technologies, Bedford, MA, US) containing 10 % heat-inactivated fetal bovine serum, 100 units/mL penicillin, and 100 units/mL streptomycin in a humid chamber at 37 °C with 5 % CO_2_. At 75 % confluence, the cells were harvested using 0.25 % trypsin, and subcultured in 75 cm^2^ flasks or six-well plates. Cells were allowed to attach to the substrate for 24 h before treatment. After serum starvation for another 24 h, four different treatments were used: (1) 10 ng/mL human recombinant TGF-β1 (Biolegend,San Diego, USA), (2) 100 µmol/L cobalt chloride (CoCl_2)_, (3) a combination of both 10 ng/ml TGF-β1 and 100 µmol/L CoCl_2_, and the same amount of PBS as for the control group. Treatment lasted for 0, 24, 36, or 48 h separately, and then cells were collected for flow cytometry and western blotting.

### Animal tumor models and treatment

NOD/SCID mice (female, 5–6 weeks old; Beijing HFK Bioscience Co. Ltd, Beijing, China) were maintained in the Laboratory for Animal Experiments of Sichuan university under specified pathogen-free conditions with animal food and water. The mice were injected subcutaneously into the right axillary fossa with A549 cells (5 × 10^6^/100 µl). When tumors were palpable and had reached approximately 500–650 mm^3^, at 7–10 days after inoculation, mice were randomly assigned into treatment and control groups with seven mice in each group. Treatments were given via tail vein injection. In this research, we utilized NS or ES for the treatment of NOD/SCID mice carrying A549 transplantation tumors for 12 days. This time point was chosen because ES has been shown to fail in tumor suppression at around this time. For the treatment (ES) group, 12.5 mg/kg ES was injected, and the control (NS) group was given the same amount of NS. The drug dosage was in accordance with the FDA human–mouse equivalent dosage conversion (FDA Guidance for Industry and Reviewers, 2002; FDA Guidance for Industry Food-Effect Bioavailability and Fed Bioequivalence studies, 2002). At day 12, all mice were euthanized, and tumors were immediately collected for flow cytometry, enzyme-linked immunosorbent assay (ELISA), and immunohistochemistry (IHC). All procedures involving animals were approved by the Animal Care and Use Committee of Sichuan University.

### Flow cytometry

The A549 tumors taken from the NOD/SCID mice were washed with ice-cold phosphate-buffered saline (PBS) pH 7.4 to remove any remaining blood, and then dissociated by mincing the tissue with scalpels, followed by addition of DMEM medium containing 1 mg/ml collagenase I and incubation for 60–90 min at 37 ℃. Incompletely dissociated tissue was digested a second time using the same procedure. The dissociated tumor tissue was then washed with ice-cold PBS and filtered through a 70-μm cell strainer (BD Bioscience). The cell suspension was then centrifuged at a speed of 1000 rpm for 5 min at 4 ℃. The cells were resuspended in PBS for further analysis.

The treated cultured A549 cells were harvested and then washed twice with ice-cold PBS.

An Aldefluor kit (StemCell Technologies, Vancouver, CA, USA) was used to analyze the population of cells with positive aldehyde dehydrogenase (ALDH) enzymatic activity. The prepared cells from both the A549 cell line and the A549 tumors were resuspended in Aldefluor assay buffer containing ALDH substrate, Bodipy™-aminoacetaldehyde (BAAA), 1 µM and incubated at 37 ℃ for 30 min according to the manufacturer’s protocol.

For assessment of CD133, CD11b, Gr1, CD206, and F4/80 expression in single cells from A549 tumors, cells were washed twice with PBS to remove remaining FBS or other impurities, and counted to make sure the cell number in each test was between 2 × 10^5^ and 1 × 10^6^. Cells were then resuspended in 100 μL PBS, and primary antibody [PE-CD133/AC133 (Miltenyi Biotec, Bergisch Gladbach, Germany), PE-Cyanine7-CD11b, APC-Gr1, or PE-F4/80 (all ebioscience, Vienna, Austria)] was added. Cells treated with CD133 antibody were placed on ice and incubated for 10 min. Cells treated with other fluorescence-labeled monoclonal antibodies were placed at room temperature in accordance with the manufacturers’ instructions, and incubated in the dark for 30 min. Cells were then rewashed with PBS and resuspended in 400–500 µl PBS, then separated in a cell sorter (FACS Aria SORP, BD Biosciences, Erembodegem, Belgium).

### Enzyme-linked immunosorbent assay

The samples collected from A549 tumors were ground into powder with liquid nitrogen in grinding bowls, and then homogenized in radioimmunoprecipitation assay (RIPA) buffer (0.1 % SDS, 0.5 % deoxycholate, 1 % Triton X-100, 150 mM NaCl, and 50 mM Tris–HCl), followed by centrifugation at 13,300 rpm for 30 min at 4 ℃. DEAB assay was used to test the protein concentration of samples. The prepared samples were stored at 80 ℃ until used. Levels of TNF-α, TGF-β, IL-6, and IL-10 in the samples were assessed by mouse ELISA kits (eBioscience or R&D Systems, Minneapolis, MN, USA) according to the manufacturer’s instructions, and the colorimetric reaction was measured at 450 nm using a microplate reader (Benchmark Electronics, Angleton, TX, USA).

### Immunohistochemical and immunoflorescence analysis

Tumors from NOD/SCID mice were immediately frozen and sliced into 5 μm sections. ALDHA1 (aldehyde dehydrogenase 1 family, member A1) expression was determined by anti-ALDHA1 antibody (Abcam) immunostaining. MVD was determined by rat anti-mouse CD31 (Biolegend) immunostaining, and TAMs were assessed by rabbit anti-mouse CD68 and rabbit anti-mouse Gr1 expression. Frozen sections were incubated in 3 % H_2_O_2_ and blocked with 5 % bovine serum albumin. The sections were then incubated with the relevant antibodies (diluted as instructions advised) at 4 ℃ overnight, followed by incubation with goat anti-rabbit or goat anti-rat antibody (diluted 1:500). Peroxidase activity was visualized using 3,3′-diaminobenzidine (DAB) substrate kit (Beyotime Bioscience, Shanghai, China). Sections were counterstained with hematoxylin. Slides were examined using a microscope (Eclipse E600; Nikon, Tokyo, Japan).

### Western blotting

A549 cells were harvested as described above. Total protein was quantified with Micro BCA Protein Assay Kit (Pierce, USA). Total protein (10 μg) from each sample was separated by electrophoresis using 12 % SDS-PAGE gels, and transferred onto PVDF membranes, blocked with 5 % skim milk (Merck), and incubated using the primary antibodies (1:1000) overnight at 4 ℃. The corresponding secondary antibodies (1:10,000) were applied for 1 h at room temperature. Glyceraldehyde 3-phophate (GAPDH) was used as a loading control. Signals were developed on X-ray films following exposure to ECL advanced luminescence.

### Statistical analysis

Data are expressed as mean ± SD. Statistical analyses were performed using the Statistical Package for the Social Sciences (version 16.0; SPSS Inc., Chicago, IL, USA). Between-group statistical significance was determined using Student’s *t* test. Significance was set at *P* < 0.05.

## Abbreviations

ES: endostatin
NS: normal saline
CSCs: cancer stem cells
LCSCs: lung CSCs
MVD: microvessel density
VEGF: vascular endothelial growth factor
TGF-β1: transforming growth factor-β1
MDSCs: myeloid-derived suppressor cells
ECs: endothelial cells
Tregs: T-regulatory cells
TAFs: tumor-associated fibroblasts
TAMs: tumor-associated microphages
HIF-1α: hypoxia-inducible factor-1α
CoCl_2_: cobalt chloride
FACS: Fluorescence Activated Cell Sorter
ALDH: aldehyde dehydrogenase
BAAA: Bodipy™-aminoacetaldehyde
GAPDH: Glyceraldehyde 3-phophate
